# The Principles and Practice of Modern Surgery

**Published:** 1844-10

**Authors:** 


### BIBLIOGRAPHICAL NOTICES.

*The Principles and Practice of Modern Surgery*, by ROBERT DRIUTT, Surgeon. Illustrated by one hundred and fifty-three wood engravings. With notes and comments by JOSHUA B. FLINT, M. D., &c.—Second American, from the 3d London edition. Philadelphia, Lea & Blanchard, 1844.—pp. 568—800. (From the Publishers.)

The issue of a second edition of the above work in this country, is a sufficient proof of the favorable reception it has met with, and the estimation in which it is held. We are happy to add that in our view this success is merited; and that the book embraces a succinct account of the principles and practice of Surgery in its present state. We can with confidence recommend it to practitioners as a work of reference, and to students as a *text book.* The engravings, which are judiciously introduced and well executed, add greatly to the practical value of the work.

